# Urinary MCP-1 and VCAM-1 as non-invasive biomarkers for the diagnosis and activity assessment of lupus nephritis

**DOI:** 10.1371/journal.pone.0323334

**Published:** 2025-05-19

**Authors:** Lichuan Lai, Chunle Wu, Xiaohua Li, Yuxiang Rong, Ying Huang, Bangqin Wang

**Affiliations:** 1 Department of Laboratory, The People’s Hospital of Guangxi Zhuang Autonomous Region, Nanning, Guangxi, China; 2 Department of Blood Transfusion, The People’s Hospital of Guangxi Zhuang Autonomous Region, Nanning, Guangxi, China; 3 Department of Rheumatology, The People’s Hospital of Guangxi Zhuang Autonomous Region, Nanning, Guangxi, China; Prime Hospital LLC, UNITED ARAB EMIRATES

## Abstract

**Introduction:**

Accurate diagnosis of lupus nephritis (LN) and effective assessment of its disease activity are essential for optimal management. This study aimed to evaluate the potential of novel urinary biomarkers, MCP-1 and VCAM-1, in diagnosing and assessing LN activity, comparing their efficacy to traditional urinary biomarkers, and proposing a new standard for clinical application.

**Methods:**

A total of 55 LN patients who met the 1997 ACR diagnostic criteria for systemic lupus erythematosus (SLE) and 34 healthy controls (HCs) were included in this study. The LN patients were categorized into two groups based on their SLE disease activity indices (SLEDAI): the inactive lupus nephritis (NALN) group (SLEDAI 0–4, n = 32) and the active lupus nephritis (ALN) group (renal SLEDAI ≥ 4, n = 22). Additionally, the patients were further classified into mild (SLEDAI 5–9), moderate (SLEDAI 10–14), and severe (SLEDAI > 14) subgroups. All LN patients underwent testing for urinary MCP-1 (uMCP-1), urinary VCAM-1 (uVCAM-1), urinary α1-microglobulin (u-α1MG), urinary β2-microglobulin (u-β2MG), urinary IgG (u-IgG), and urinary albumin (u-ALB), as well as a percutaneous renal biopsy.

**Results:**

The levels of urinary MCP-1 and VCAM-1 (uMCP-1 and uVCAM-1) in the LN group were significantly elevated compared to the HCs (uMCP-1: *P* < 0.001; uVCAM-1: *P* < 0.01). Receiver operating characteristic (ROC) curve analysis revealed that the diagnostic efficacy of uMCP-1 and uVCAM-1 surpassed that of traditional biomarkers (uMCP-1: AUC = 0.79, *P* < 0.001; uVCAM-1: AUC = 0.77, *P* < 0.001). Multivariate logistic regression demonstrated a significant association between uMCP-1 and uVCAM-1 levels and the occurrence of LN (*P* < 0.001). Furthermore, these novel biomarkers exhibited stronger correlations with SLEDAI scores than traditional biomarkers (P < 0.001). Notably, patients with ALN had significantly higher levels of uMCP-1 and uVCAM-1 compared to those with NALN (uMCP-1: *P* < 0.01; uVCAM-1: *P* < 0.01).

**Conclusion:**

The production of uMCP-1 and uVCAM-1 is closely associated with the onset and progression of LN (ISN/RPS: Class I - IV). These biomarkers may serve as valuable references for the diagnosis and prediction of LN and aid in the assessment of LN activity.

## Introduction

Systemic lupus erythematosus (SLE) is a chronic autoimmune disease that ultimately leads to damage in various organs and tissues [1]. The kidneys are one of the primary target organs in SLE patients, with approximately 50–80% of patients experiencing renal complications, referred to as lupus nephritis (LN), a leading cause of clinical symptoms and mortality in SLE patients [2,3]. The pathogenesis of LN is complex, involving factors such as complement activation, cell apoptosis, and genetic susceptibility, eventually resulting in loss of self-tolerance and the synthesis of numerous cytokines and chemokines by renal structural and immune cells, thereby promoting the development and progression of LN [4–6]. The course of LN is prolonged, characterized by fluctuating symptoms and poor prognosis, with a significant proportion of patients progressing to end-stage renal disease within 5 years of onset [7]. Therefore, long-term follow-up monitoring of LN patients is critical in the clinical setting to assess disease progression and treatment response. At present, renal biopsy remains the gold standard for diagnosing LN pathologically, providing valuable information on the degree of involvement of renal glomeruli, tubules, vessels, and interstitium [8,9]. Indeed, it plays a pivotal role in evaluating the disease stage and response to therapy [10,11]. However, renal biopsy is an invasive procedure associated with complications, making frequent repetition impractical. Hence, there is an urgent need to develop non-invasive biomarkers to diagnose and predict the activity of LN.

Recently, an increasing number of international studies have reported that substances detectable in serum or urine samples have the potential to serve as biomarkers for the clinical diagnosis and prognostic prediction of LN [12–15]. Among these, urine, which is directly filtered by the kidneys and non-invasively collected, offers a greater advantage in identifying LN biomarkers. Currently, commonly used urinary biomarkers for LN include urinary alpha 1-microglobulin (u-α1MG), urinary-beta2-microglobulin (u-β2MG), urinary immunoglobulin G (u-IgG), and urinary microalbumin (u-ALB) [16–19]. Notwithstanding, the reliability of these traditional indicators for the diagnosis of LN remains suboptimal, and they have no significant advantage in assessing disease activity [20,21]. With advances in animal models and proteomic sequencing techniques, pro-inflammatory cytokines have progressively emerged as novel biomarkers for evaluating LN-induced renal damage, among which monocyte chemoattractant protein-1 (MCP-1) and vascular cell adhesion molecule-1 (VCAM-1) have garnered extensive attention [22,23]. The former, a member of the chemokine family, is generated by various cell types and highly expressed in activated monocytes/macrophages, T cells, and natural killer cells. Additionally, it mediates immune cell infiltration in the kidneys [24]. Previous studies have shown that uMCP-1 levels are significantly elevated in patients with active lupus nephritis (ALN) and demonstrate superior sensitivity and specificity in detecting lupus activity and LN activity [25,26]. Furthermore, uMCP-1 is correlated with proteinuria and renal SLEDAI-2K, playing an important role in monitoring disease activity and treatment response [27,28]. On the other hand, the latter is a cell adhesion molecule expressed on the surface of various cells, induced in endothelial cells in response to inflammatory cytokines (including tumor necrosis factor and interleukin-1), and interacts with integrins (very late antigen-4) on infiltrating leukocytes to facilitate the migration and recruitment of inflammatory cells [29]. VCAM-1 serves as a crucial marker of inflammatory response in SLE and is closely associated with endothelial dysfunction and the disease’s immune mechanisms. Additionally, it may play a vital role in monitoring disease progression and evaluating prognosis [30,31]. Of note, their characteristics appear to be closely associated with renal inflammation and thus may serve as indicators of kidney injury.

However, studies comparing MCP-1 and VCAM-1 with traditional urinary biomarkers are scarce. Therefore, this study aimed to identify differences between novel urinary biomarkers (i.e., uMCP-1 and uVCAM-1) and traditional LN urinary biomarkers (i.e., u-α1MG, u-β2MG, u-IgG, u-ALB) in LN patients and healthy individuals to determine their diagnostic efficacy for LN and for assessing LN activity.

## Materials and methods

### Study design and participants

This study conducted at the People’s Hospital of Guangxi Zhuang Autonomous Region. Between June 2023 and December 2023, 55 LN patients scheduled to receive treatment in the rheumatology department were randomly selected for this study. During the same period, 34 healthy individuals undergoing physical examination at the same hospital were enrolled as Healthy Controls (HCs). HCs were age- and sex-matched healthy subjects with normal blood pressure and urine analysis results. This study received ethical approval from the People’s Hospital of Guangxi Zhuang Autonomous Region Ethics Committee (Approval No: KY-ZC-2022–162), with all participants providing written informed consent prior to inclusion in the research.

u-α1MG, u-β2MG, u-IgG, and u-ALB levels were measured as routine laboratory tests, with data collected from electronic medical records. The reporting of this study conforms to STROBE [32].

### Inclusion criteria

(1) Aged ≥ 18 years and fulfilling the revised 1997 classification criteria of the American College of Rheumatology (ACR) for SLE [33]; (2) Based on the results of pathological puncture, and The International Society of Nephrology/Renal Pathology Society (ISN/RPS) criteria were used to diagnosed LN [34].

### Exclusion criteria

Drug-induced lupus, active malignancy, overlap syndrome, end-stage renal disease due to non-SLE cause. Clinical information, including age, gender, and clinical manifestations, was collected.

### Sociodemographic and clinical assessment

All patients and controls underwent routine laboratory tests, including complete blood count, blood urea, serum creatinine, complement C3 (C3), complement C4 (C4), erythrocyte sedimentation rate (ESR), C-reactive protein, liver function tests, urinalysis, and 24-hour urine protein quantification.

### Laboratory assessments

Morning midstream urine samples were collected in sterile containers and centrifuged at 3000 rpm for 10 minutes to remove debris. Next, the supernatants were aliquoted and stored at -80°C for the ensuing analysis. Following this, the levels of traditional urinary biomarkers (u-α1MG, u-β2MG, u-IgG, u-ALB) were measured using a Beckman AU-5800 automatic biochemical analyzer. This experiment was conducted in the Department of Laboratory at the People’s Hospital of Guangxi Zhuang Autonomous Region following standard laboratory procedures. The laboratory is an accredited facility recognized under ISO 15189 standards.

### Assessment of disease activity

The SLE Disease Activity Index (SLEDAI) was used to assess disease activity [35]. The renal SLEDAI (rSLEDAI) provides a sum score for the renal domains of SLEDAI, including hematuria (>5 red blood cells/high-power field), pyuria (>5 white blood cells/high-power field), proteinuria (>0.5 g/24 h), and urinary casts. LN patients were classified into the non-active LN (NALN) group (SLEDAI 0–4, n = 32) and the active LN (ALN) group (rSLEDAI ≥ 4, n = 22) which was further stratified into mild (SLEDAI 5–9), moderate (SLEDAI 10–14), and severe (SLEDAI > 14) subgroups.

### Measurement of urinary MCP-1 and VCAM-1

Urinary MCP-1 and VCAM-1 levels were quantified using enzyme-linked immunosorbent assay (ELISA) kits according to the manufacturer’s instructions. Briefly, MCP-1 and VCAM-1 antibodies were pre-coated onto different wells. Samples, standards, and HRP-labeled detection antibodies were added, followed by incubation at 37°C for 60 minutes. After five washes, substrate solution was introduced, and the plates were incubated in the dark for 15 minutes. Then, the reaction was terminated, and the optical density of MCP-1 and VCAM-1 was measured at 450 nm using a microplate reader. A standard curve was plotted to calculate sample concentrations. The minimum detection limit of the kit was 1.0 ng/mL for both MCP-1 and VCAM-1. Urine samples for uMCP-1 and uVCAM-1 were diluted 1:5 as required.

### Establish a multivariate logistic regression analysis model

Next, logistic regression was employed to analyze the impact of various factors on the risk of developing LN. Data on relevant variables, including age, as well as the levels of uMCP-1, uVCAM-1, u-β2MG, u-IgG, and u-ALB, were collected and used as input features, with the presence or absence of LN serving as the output variable. Concentrations of urinary biomarkers are detailed in S1 Appendix.

### Statistical analysis

Statistical analyses and visualization of date were performed using GraphPad Prism v.9.5.1 (GraphPad, San Diego, CA, USA). The t-test was used for group comparisons, and Pearson’s correlation analysis was also performed. The mean and standard deviation of continuous, normally distributed data were presented (SD). The concentration of urinary biomarkers has been standardized using urine creatinine concentration. A *P-*value less than 0.05 was considered statistically significant.

## Results

### Comparison of patient characteristics

A total of fifty-five LN patients were included (87.27% females), with a mean age of 39.30 ± 14.70 years. The median SLEDAI score of patients was 10, ranging from 0 to 17. According to the SLEDAI activity index, 32 cases (58.00%) and 22 cases (42.00%) were assigned to the NALN group and the ALN group, respectively, with 10 cases (45.45%) classified as mild, 9 cases (40.09%) as moderate, and 3 cases (13.63%) as severe. As controls, thirty-four healthy subjects were included in the HCs group, comprising 61.77% females, with a mean age of 50.30 ± 13.51 years.

### Application of uMCP-1 and uVCAM-1 in distinguishing LN patients from HCs

**Fig 1:**
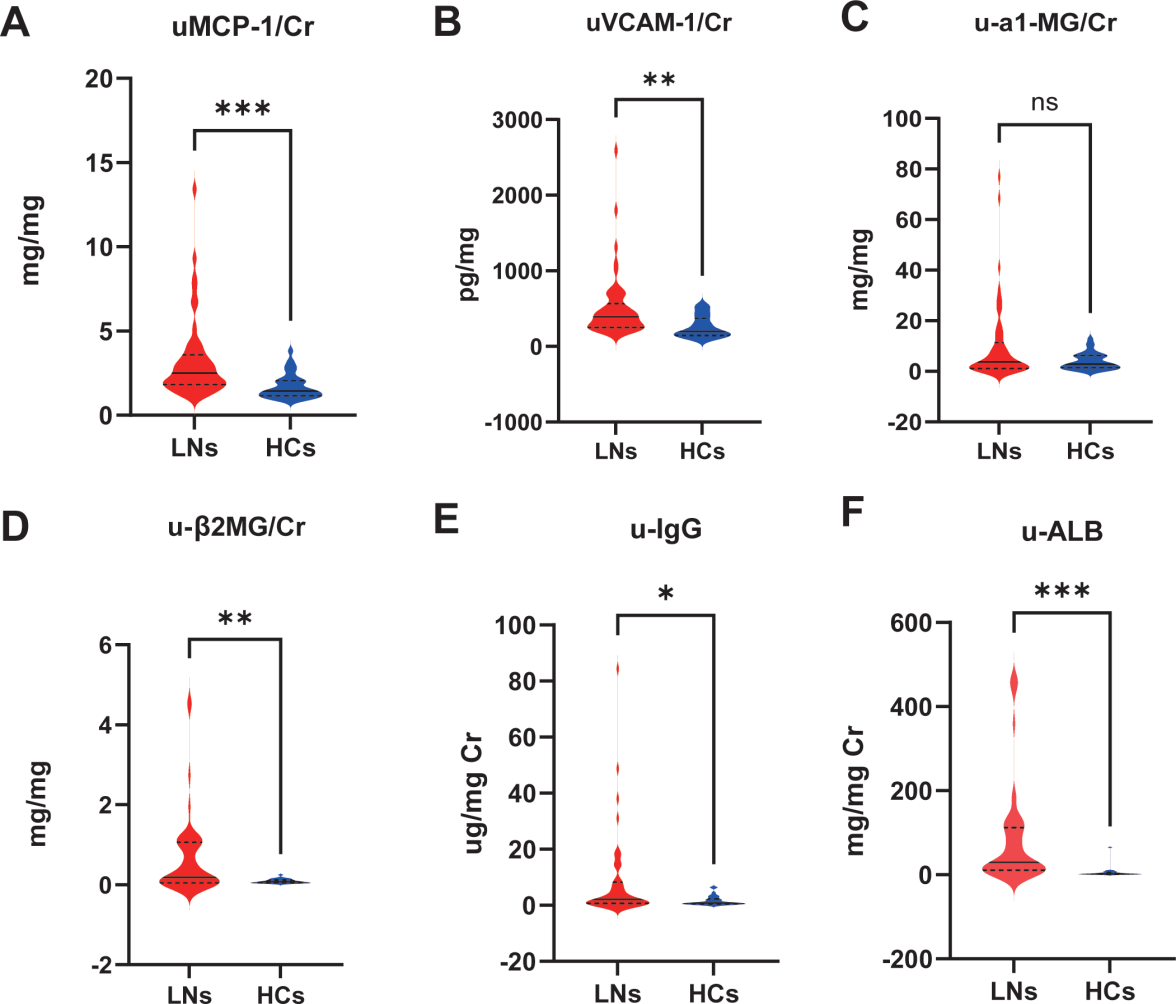
Novel urinary biomarker and traditional urinary biomarker in distinguishing LN patients from HCs. **(A)** The difference between the levels of uMCP-1 in LNs and HCs. **(B)** The difference between the levels of uVCAM-1 in LNs and HCs. **(C)** The difference between the levels of u-α1MG in LNs and HCs. **(D)** The difference between the levels of u-β2MG in LNs and HCs. **(E)** The difference between the levels of u-IgG in LNs and HCs. **(F)** The difference between the levels of u-ALB in LNs and HCs. *P < 0.05, **P < 0.01, ***P < 0.001.

The results revealed that uMCP-1/creatinine in urine (uMCP-1/Cr) and uVCAM-1/creatinine in urine (uMCP-1/Cr) were significantly elevated in the LN group (uMCP-1/Cr, LN verse HCs, 3.242 ± 0.340 verse 1.693 ± 0.123, *P* ＜ 0.001; uVCAM-1/Cr, LN verse HCs, 511.5 ± 60.95 vs 257.5 ± 24.53, *P* ＜ 0.01) (Fig 1A and 1B).

Regarding traditional biomarkers, the difference in u-a1MG was not statistically significant (*P* > 0.05) (Fig1 C). However, the levels of u-β2MG, u-IgG, and u-ALB were noticeably higher in the LN group (u-β2MG/Cr, LN verse HCs, 0.18 (0.04–1.06) verse 0.07 (0.44–0.11), *P* ＜ 0.01; u-IgG/Cr, LN verse HCs, 2.19 (0.63–8.27) verse 0.88 (0.52–2.24), *P* ＜ 0.05; u-ALB/Cr, LN verse HCs, 29.98 (11.07–112.6) verse 2.26 (1.208–4.92), *P* ＜ 0.001) (Fig 1D - F).

**Fig 2:**
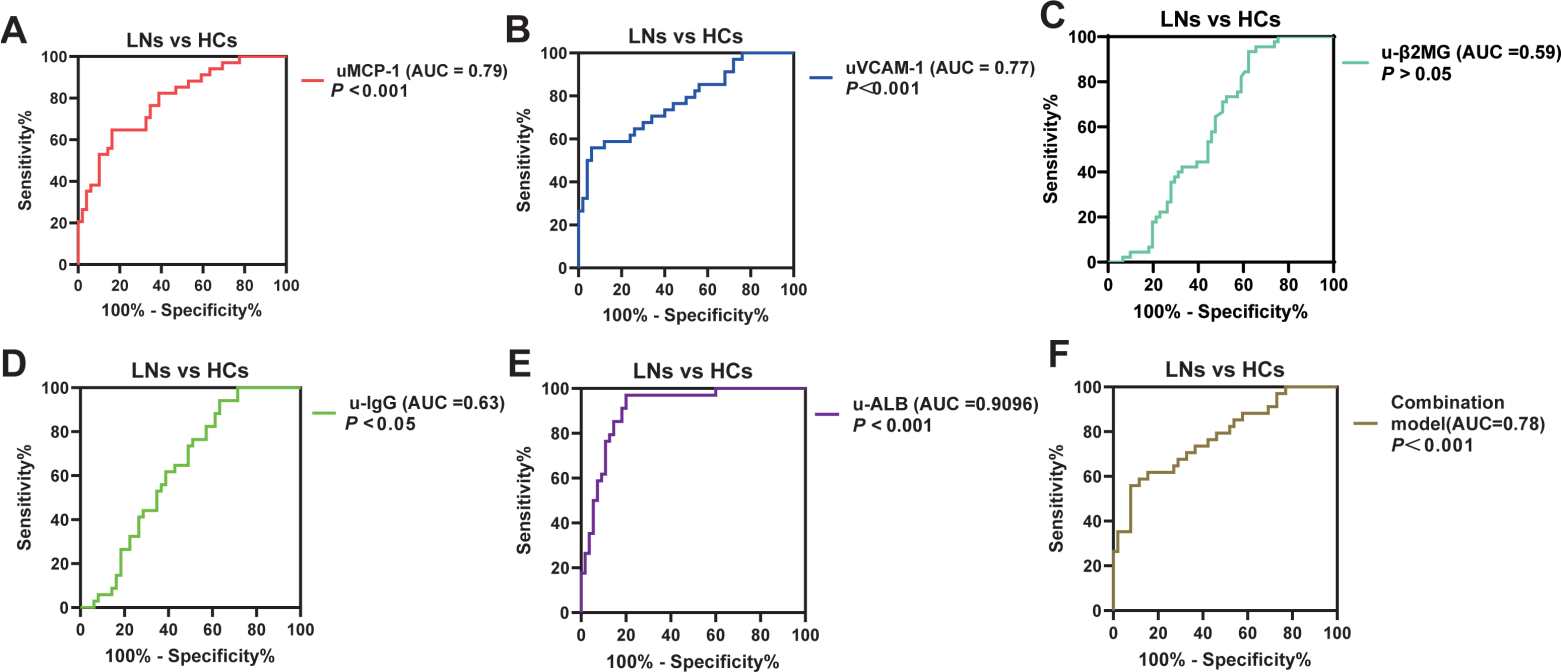
ROC curves were used to evaluate the diagnostic performance in patients with LN. The AUC value for (A) uMCP-1 and (B) uVCAM-1 were significantly higher compared to (C) u-β2MG, (D) u-IgG and (E) u-ALB. By combining uMCP-1 and uVCAM-1 using logistic regression, the combination model’s AUC(Y = 0.003*uMCP-1* + 0.002u*VACM-1–1.209) increased to 0.94 (*P* < 0.0001), demonstrating it as an excellent multivariate diagnostic model for clinical LN. *P < 0.05, **P < 0.01, ***P < 0.001.

Subsequently, ROC curves were plotted to evaluate their diagnostic performance for LN (Fig 2). As anticipated, the results demonstrated that the diagnostic value of uMCP-1/Cr and uVCAM-1/Cr (The AUC (95% CI) of them is 0.79(0.69,0.88) and 0.77(0.67,0.87)) was superior to that of u-β2MG/Cr (AUC (95% CI)=0.59 (0.46, 0.70)), u-IgG (AUC (95% CI)=0.63 (0.51, 0.74)), but lower than that of u-ALB/Cr (AUC (95% CI)=0.90 (0.84, 0.97)). Meanwhile, the sensitivity, specificity, and LR+ of uMCP-1 (26.47%, 97.96%, and 12.97, respectively) and uVCAM-1/Cr (32.35%, 98.00%, and 16.18, respectively) were higher than those of traditional urine markers (Fig 2A - E). Additionally, a combined diagnostic model was incorporated uMCP-1 and uVCAM-1,the model represented by the equation *Y* = 0.003*uMCP/Cr -1 + 0.002u*VACM/Cr -1–1.209, which achieved a higher AUC (AUC (95% CI = 0.77 (0.67, 0.87)), sensitivity (35.29%), specificity (98.08%), and LR+ (18.35) (Fig 2F).

In the multivariate logistic regression model incorporating age, uMCP-1, uVCAM-1, u-β2MG, u-IgG, and u-ALB, the results exposed that after adjusting for age, uMCP-1 was significantly associated with the incidence of LN (uMCP-1, OR = 6.99, 95% CI 1.19–41.131, *P* = 0.031;). In contrast, u-β2MG, and u-IgG were not significantly associated with LN incidence (u-β2MG, OR = 4.84, 95% CI 0.249–94.373, *P* = 0.298; u-IgG, OR = 0.98, 95% CI 0.72–1.35, *P* = 0.918;), as presented in Table 1. Overall, uMCP-1 was positively correlated with LN, signifying that higher level was associated with a higher likelihood of disease occurrence. However, traditional urine biomarkers (u-β2MG and u-IgG) did not significantly predict disease occurrence, indicating that uMCP-1 and uVCAM-1 can serve as independent predictors of LN.

**Table 1:** The information on the multivariate logistic regression in the presented study.

Dependent: LNs		LNs (N = 55)	HCs (N = 34)	OR (multivariable)
Age	Mean ± SD	39.3 ± 14.7	50.3 ± 13.5	0.94 (0.88–1.01, *P* = 0.33)
uMCP-1/Cr	Mean ± SD	3.242 ± 0.340	1.693 ± 0.123	6.99 (1.19–41.1, *P* = 0.031)
uVCAM-1/Cr	Mean ± SD	511.5 ± 60.95	257.5 ± 24.53	1..00 (0.99–1.01, *P* = 0.67)
u-β2MG/Cr	Median(P25, P75)	0.18 (0.04-1.06)	0.07 (0.44-0.11)	4.85 (0.25–94.37, *P* = 0.30)
u-IgG/Cr	Median(P25, P75)	2.19 (0.63- 8.27)	0.88 (0.52-2.24)	0.99 (0.72–1.35, *P* = 0.94)
u-ALB/Cr	Median(P25,P75)	29.98 (11.07-112.6)	2.26 (1.208-4.92)	1.10 (1.02–1.19, *P* = 0.02)

### Comparison of lupus disease activity-related markers

**Fig 3:**
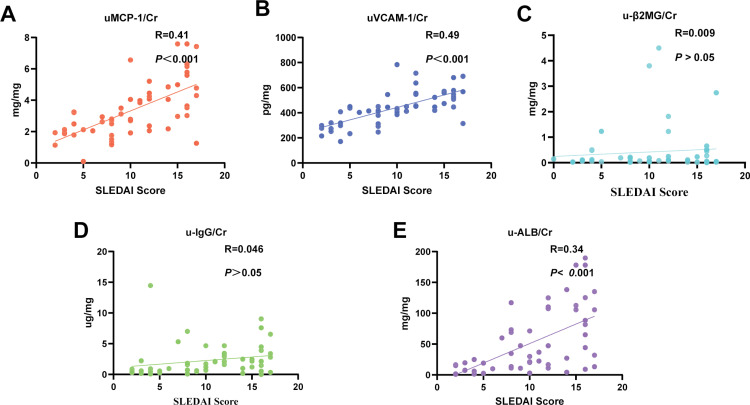
Correlation of novel urinary biomarker and traditional urinary biomarker with SLEDAI score. (A) uMCP-1 shows correlation with score (R = 0.41). (B) uVCAM-1 shows correlation with score (R = 0.49). (C) u-β2MG shows correlation with score (R = 0.009). (D) u-IgG shows correlation with score (R = 0.46). (E) u-ALB shows correlation with score (R = 0.11). *P < 0.05, **P < 0.01, ***P < 0.001.

The SLEDAI score is commonly used to assess disease activity [35]. Spearman rank correlation tests showed determined that uMCP-1 and uVCAM-1 were considerably correlated with SLEDAI scores (uMCP-1, R = 0.41, *P* ＜ 0.001; uVCAM-1, R = 0.49, *P* ＜ 0.001) and demonstrated a superior correlation compared to traditional markers (u-β2MG, R = 0.009, *P* > 0.05; u-IgG, R = 0.46, *P* > 0.05; u-ALB, R = 0.34, *P* < 0.05)(Fig 3). Furthermore, we also calculated the concentrations of MCP and VCAM for each pathological type of LN, as well as the activity scores. (Table 2)

**Table 2:** Pathological classification-related data of LN patients. (Mean ± SD).

ISN/RPS classification	u-MCP-1/Cr	u-VCAM-1/Cr	Activity index (0–17)
Class I (N = 12)	4.87 ± 3.99	322.5 ± 150.5	7.75 ± 4.288
Class II (N = 15)	2.97 ± 1.66	373.2 ± 199.5	8.73 ± 3.56
Class III (N = 16)	1.77 ± 0.64	279.6 ± 106.2	8.77 ± 4.09
Class IV (N = 12)	2.59 ± 1.64	484.3 ± 211.4	15.00 ± 2.34

**Fig 4:**
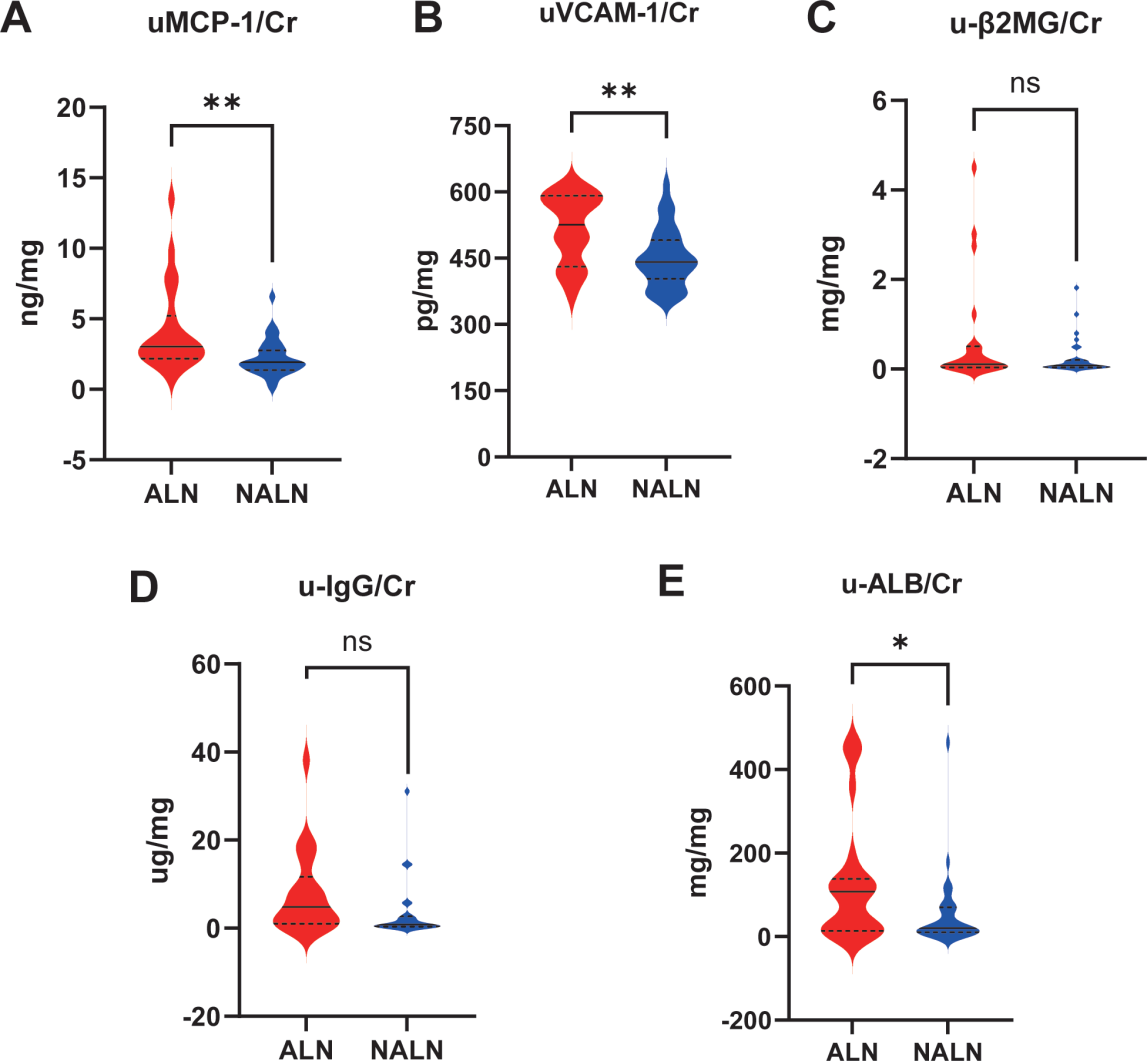
The utility of novel urinary biomarker and traditional urinary biomarker in evaluating lupus disease activity. **(A)** The difference between the levels of uMCP-1 in ALN and NALN. **(B)** The difference between the levels of uVCAM-1 in ALN and NALN. **(C)** The difference between the levels of u-β2MG in ALN and NALN. **(D)** The difference between the levels of u-IgG in ALN and NALN. **(E)** The difference between the levels of u-ALB in ALN and NALN. *P < 0.05, **P < 0.01, ***P < 0.001.

Compared to the NALN group, the levels of uMCP-1/Cr and uVCAM-1/Cr were significantly higher in the ALN group (uMCP-1/Cr, ALN group vs NALN group, 4.161 ± 3.077 vs 2.150 ± 1.250, *P < *0.0001; uVCAM-1/Cr, ALN group vs NALN group, 517.4 ± 78.07 vs 450.7 ± 67.01, *P <* 0.01). In contrast, the levels of traditional LN biomarkers were comparable between the ALN and NALN groups (Fig 4), implying that uMCP-1 and uVCAM-1 were significantly associated with disease activity, whereas the traditional clinical LN biomarkers u-β2MG, u-IgG, and u-ALB were not.

**Fig 5:**
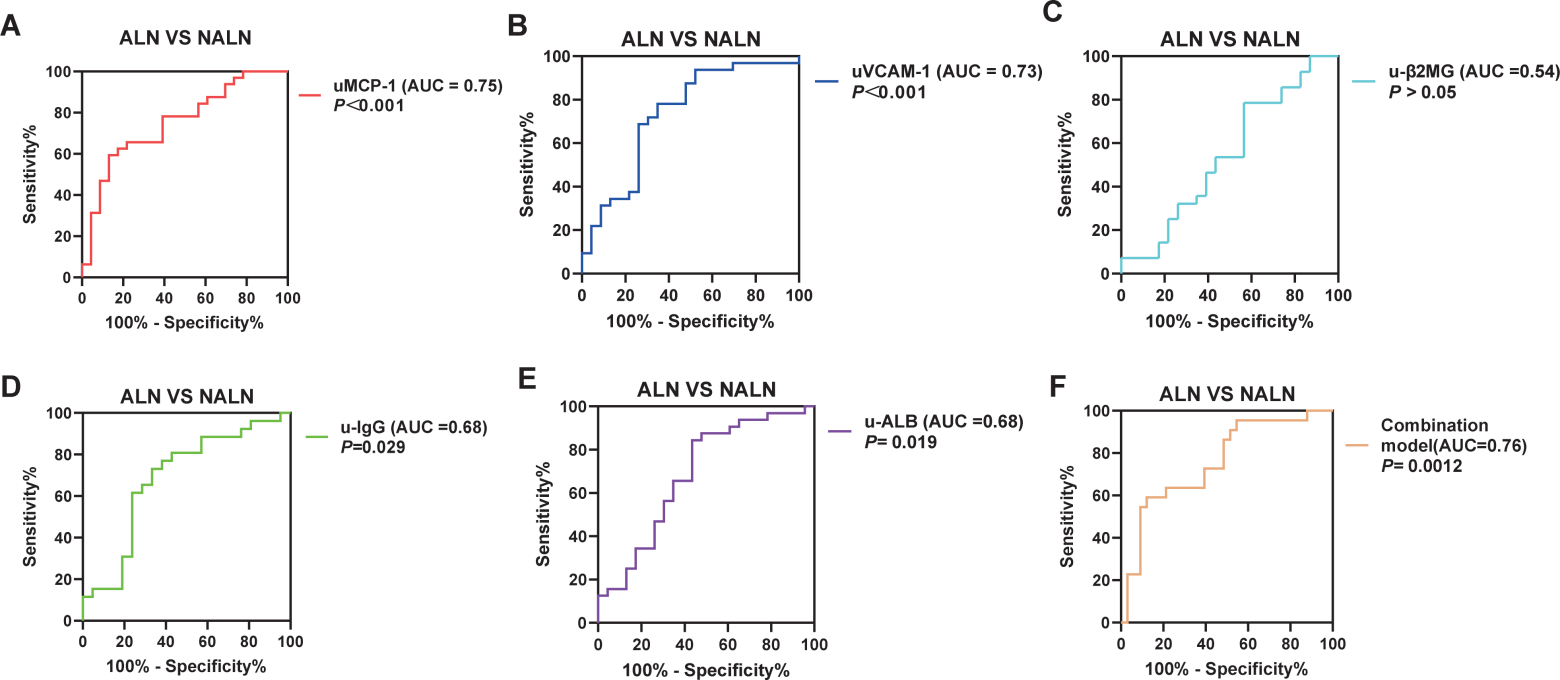
ROC curves were used to assess the discriminative ability for clinical lupus disease activity in patients. The AUC value for (A) uMCP-1 and (B) uVCAM-1 were significantly higher than those for (C) u-β2MG, (D) u-IgG and (E) u-ALB. The previously constructed combination model (Y = 0.003*uMCP-1* + 0.002u*VACM-1–1.209) also demonstrated strong discriminative power for distinguishing between ALN and NALN. *P < 0.05, **P < 0.01, ***P < 0.001.

ROC curves were plotted to further examine the diagnostic value for LN activity. The results showed that the diagnostic performance of uMCP-1/Cr (AUC (95% CI)=0.77 (0.64,0.89), *P <* 0.001) and uVCAM-1/Cr (AUC (95% CI)=0.73 (0.59, 0.87), *P <* 0.01) was higher than that of u-β2MG (AUC (95% CI)=0.54 (0.38, 0.70), *P* > 0.05), u-IgG/Cr (AUC (95%CI)=0.68 (0.52, 0.84), *P <* 0.05), and u-ALB/Cr (AUC (95% CI)=0.68 (0.53, 0.83), *P <* 0.05)(Fig 5A-E). When individually assessed, uMCP-1/Cr exhibited higher sensitivity (31.25%) and specificity (95.65%) compared to uVCAM-1/Cr (sensitivity 21.88%, specificity (95.65%), indicating that uMCP-1/Cr and uVCAM-1/Cr may be more effective biomarkers for assessing LN activity. At the same time, the combined diagnostic model incorporating both uMCP-1/Cr and uVCAM-1/Cr had higher AUC (AUC (95% CI)=0.75 (0.63, 0.88), *P* ＜ 0.01), sensitivity (22.73%), specificity (96.97%), and LR+ (7.5) compared to their individual parameters (Fig 5F).

**Fig 6:**
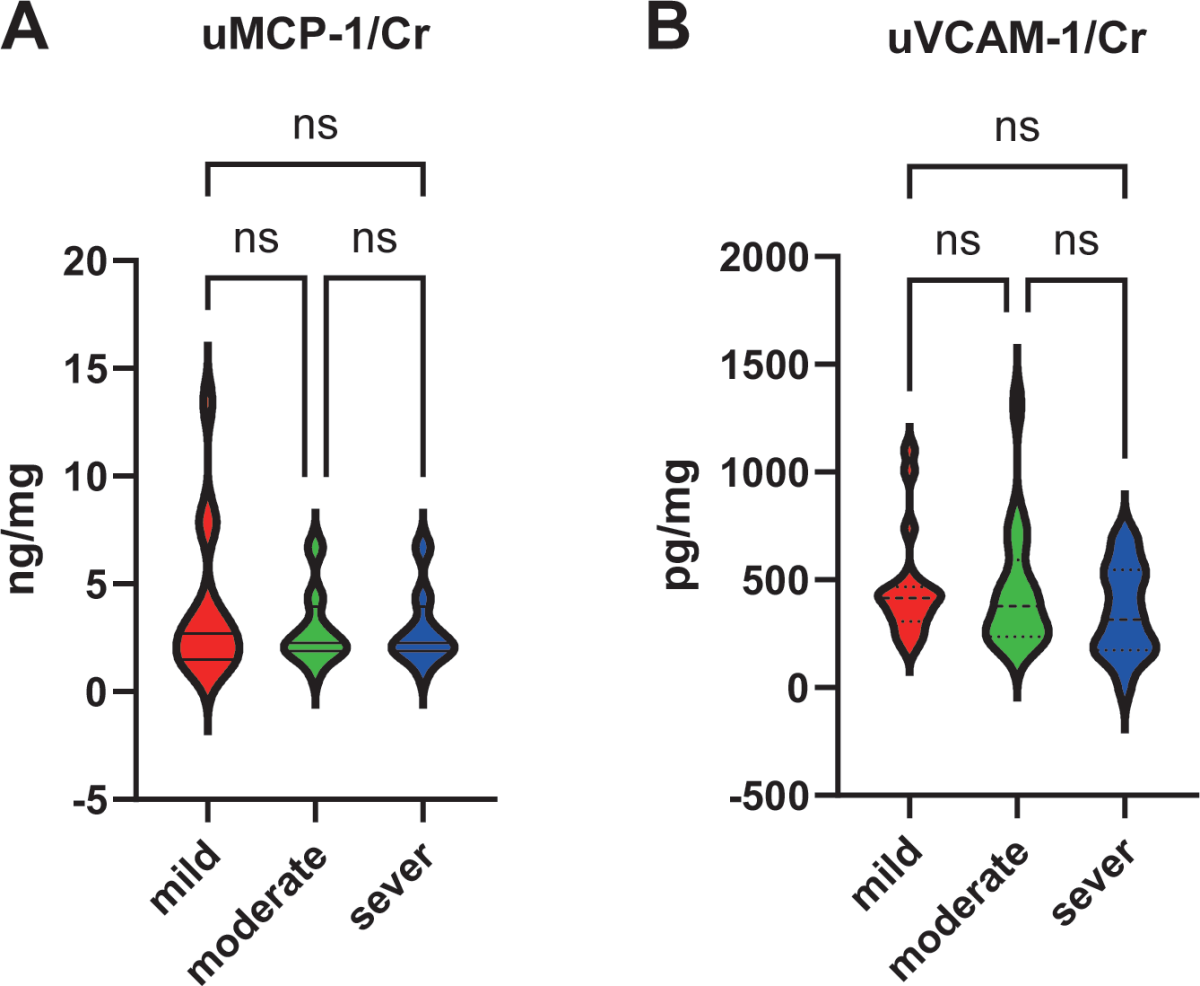
Association of uMCP-1 and uVCAM-1 with LN disease severity. **(A)** The relationship between uMCP-1 and mild, moderate, and severe activity groups (*P* > 0.05); **(B)** The relationship between uVCAM-1 and mild, moderate, and severe activity groups (*P* > 0.05).

To further validate the relationship between uMCP-1, uVCAM-1, and disease activity, we compared different parameters among patients stratified into the mild, moderate, and severe activity groups. The results uncovered no substantially differences in uMCP-1 and uVCAM-1 across the activity levels (*P* > 0.05), indicating that they could not accurately differentiate between the different activity levels of LN patients (Fig 6).

## Discussion

Renal involvement is a frequent and serious complication of SLE [36,37]. Initially, LN was understood mainly through renal pathology and clinical symptoms. However, extensive research over the years into its pathology, clinical features, and biology has deepened our understanding of LN’s pathophysiology [38]. Despite these advances, renal biopsy remains the gold standard for diagnosing and assessing LN activity, although its invasive nature and potential complications present significant drawbacks [39], not all hospitals can readily perform renal biopsy for diagnosis. Therefore, this study shifted its focus to identifying non-invasive biomarkers to serve as more accurate and sensitive indicators of the disease. In recent years, cytokines and chemokines have been increasingly recognized for their critical roles in the pathogenesis and diagnosis of renal injury [40,41]. MCP-1 and VCAM-1, as non-invasive markers, play an essential role in LN that cannot be overlooked. They not only have diagnostic potential but also provide valuable insights into disease activity and prognosis [15,42].

On one hand, MCP-1 is predominantly produced by renal tubular epithelial cells, interstitial cells, and glomerular endothelial cells. In the kidneys of LN mouse models and human inflammatory renal diseases, cell apoptosis induced by the Fenton reaction has been observed, which promoted cell membrane damage and subsequent renal cell death [43]. Furthermore, oxidative stress in cells induces NF-ĸB activation in glomerular endothelial cells, which, in turn, up-regulates MCP-1 expression [44–46]. On the other hand, VCAM-1 is a key adhesion molecule that plays a pivotal role in the transportation of inflammatory cells and lymphocytes. Its mechanism of action principally involves interaction with integrins on the surface of leukocytes, thereby driving the recruitment and infiltration of inflammatory cells [47]. Leukocyte infiltration is typically regarded as a significant hallmark of kidney diseases and a typical manifestation of renal inflammation. During this process, the expression level of VCAM-1 increases, especially in renal tubular epithelial cells and glomerular endothelial cells, thereby affecting glomerular filtration, vascular permeability, and kidney function [48–50].

It is worthwhile emphasizing that limited studies compared uMCP-1 and uVCAM-1 with traditional biomarkers or explored their combined diagnostic potential. Herein, uMCP-1 and uVCAM-1 had superior diagnostic potential for LN than traditional markers (u-α1MG, u-β2MG and u-IgG). Likewise, uMCP-1 and uVCAM-1 were superior in distinguishing ALN from NALN and were closely related to SLEDAI scores, consistent with the observation of previous studies [51–53]. Additionally, to explore the significance of a multi-index approach for the diagnosis of LN, the diagnostic value of the combination of uMCP-1 and uVCAM-1 was investigated, unveiling that this combination significantly enhanced the diagnostic accuracy for LN and assessment of LN activity.

Traditional urinary biomarkers such as u-α1MG, u-β2MG, and u-ALB are characterized by their low molecular weight, allowing them to pass through the glomerulus and be largely reabsorbed by proximal epithelial cells. Studies undertaken by Stefanovic, Kaysen, and Jihua et al. [54–56] have demonstrated that the levels of these biomarkers are significantly elevated in patients with glomerulonephritis and interstitial nephritis. Furthermore, Woong et al. [57] evinced that u-IgG, as a key substance implicated in the formation of immune complexes in the subendothelial and subepithelial regions of the glomerulus, was significantly elevated in patients with LN and membranous nephropathy. Herein, our results indicated that u-α1MG may not be suitable for reflecting LN status. While u-β2MG, u-IgG, and u-ALB can distinguish LN status, they cannot accurately delineate the LN phase, as evidenced by their weak correlation with SLEDAI scores (u-β2MG, R = 0009, *P* > 0.05; u-IgG, R = 0.46, *P* > 0.05; u-ALB, R = 0.34, *P* < 0.05). Consequently, they displayed limited efficacy in identifying active lupus patients. Our results showed that uMCP-1 and uVCAM-1 were significantly correlated with the SLEDAI score (uMCP-1/Cr, R = 0.41, P < 0.001; uVCAM-1/Cr, R = 0.49, P < 0.001), suggesting that higher concentrations are associated with a greater likelihood of lupus being in an active phase. It is well established that α1MG, β2MG, ALB, and IgG are primarily derived from blood cells and are not strongly correlated with renal cells. As urinary biomarkers, they do not effectively reflect inflammatory infiltration in the kidneys [36,58,59]. Therefore, the cellular characteristics of uMCP-1 and uVCAM-1 enable them to better reflect LN and its activity compared to traditional urinary biomarkers.

Furthermore, ROC curve analysis illustrated that the diagnostic potential of uMCP-1 and uVCAM-1 for LN exceeded that of traditional urinary biomarkers (uMCP-1, AUC = 0.78, uVCAM-1, AUC = 0.77; vs u-β2MG, AUC = 0.58, u-ALB, AUC = 0.90, u-IgG, AUC = 0.63). More importantly, logistic regression analysis demonstrated that the predictive value of uMCP-1 and uVCAM-1 was higher compared to traditional urinary biomarkers, establishing them as independent predictors of LN. In evaluating LN activity, uMCP-1 and uVCAM-1 also outperformed traditional urinary biomarkers in distinguishing ALN (rSLEDAI score ≥ 4) from inactive LN (SLEDAI score ≤ 4) (uMCP-1, AUC = 0.75, *P* < 0.01; uVCAM-1, AUC = 0.73, *P* < 0.01). Besides, the combination of uMCP-1 and uVCAM-1 possessed outstanding performance in both diagnosing LN and evaluating LN activity, with AUC of 0.78 and 0.76, respectively, exceeding the performance of individual biomarkers. uMCP-1 and uVCAM-1 showed superior diagnostic efficacy in our study, which is consistent with previous studies [23,25,26]. The combination model also exhibited excellent sensitivity, specificity, and LR + . Although the model lacked specificity in diagnosing LN and sensitivity in assessing LN activity, it can serve as an auxiliary tool to kidney biopsy in cases where biopsy is not feasible, potentially enhancing the diagnosis of LN and the evaluation of LN activity.

Finally, the potential of uMCP-1 and uVCAM-1 to differentiate the severity levels of disease activity was investigated. The findings showed that they could not effectively distinguish between patients in the mild, moderate, and severe subgroups of the active group. This result differs from that of previous studies [48,60,61]. However, we postulate that this discrepancy may be ascribed to the relatively small sample size in our study, which could have contributed to the lack of significant correlation in further differentiating the degrees of activity using uMCP-1 and uVCAM-1.

In summary, our study results indicate that uMCP-1 and uVCAM-1 are superior to traditional urinary biomarkers in diagnosing LN and are highly effective in distinguishing LN activity. However, the research has several limitations. First, the classification of LN activity is based on SLEDAI, which may overlook other important renal pathological changes and cannot fully reflect variations in LN activity. Second, our cohort of LN patients lacks those with Class V pathology, limiting our conclusions to patients with Class I - IV. Third, we only compared the biomarker levels of LN patients with healthy individuals, lacking comparisons with other SLE patients. Fourth, this is a cross-sectional study without longitudinal follow-up, and the relatively small sample size may affect the interpretation of the results. Therefore, larger, multi-center prospective studies are needed to validate the diagnostic efficacy of uMCP-1 and uVCAM-1 and to further explore their clinical applicability. Fifth, the effect of the drug on the outcomes was not assessed. Additionally, we aim to assess the specificity of uMCP-1 and uVCAM-1 for LN. Although our findings indicate elevated serum IL-6 levels in LN patients, we cannot exclude the possibility that other types of inflammatory or non-inflammatory renal diseases may also elevate these levels.

In future studies, further experimental support will be necessary, including the detection of serum MCP-1 and VCAM-1 in patients, as well as histochemical experiments on renal tissue. Additionally, follow-ups can be conducted to assess the levels of patients before and after treatment.

## Conclusion

This study demonstrates that the novel urinary biomarkers uMCP-1 and uVCAM-1 not only have the potential to diagnose LN (ISN/RPS: class I to IV), but their levels also show a significant correlation with SLEDAI activity scores and possess predictive value for disease onset. Moreover, compared to traditional urinary biomarkers such as u-β2MG, u-IgG, and u-ALB, uMCP-1 and uVCAM-1 demonstrated greater diagnostic efficacy and correlation with SLEDAI scores. Therefore, u-MCP1 and u-VCAM1 may represent a promising approach for diagnosing LN and evaluating LN activity.

## Supporting information

S1 Appendix (XLSX)
